# On a "Black Fur on the Tongue."

**Published:** 1854-07

**Authors:** 

**Affiliations:** Coblenz.


### ARTICLE XVII.

*On a "Black Fur on the Tongue."* By DR. EULENBERG, of Coblenz. Abstracted from the 'Arch. f. Physiol. Heilk.,' August, 1853.

THE author relates, that in the preceding year a child, two years old, was brought to him, whose tongue was covered with a perfectly black coating. The organ, from the tip to the back, appeared as if it were smeared with ink; and at first sight, the supposition necessarily entertained was, that the child had licked some black object, or had swallowed a colored liquid. Except a slight diarrhœa, the boy presented no other morbid symptoms. For his age, he was well developed, and had never had any important illness. The author's immediate treatment was confined to washing the tongue with vinegar and water.

Fourteen days afterwards, the child was again brought to him, when the mother stated that the washing of the tongue had removed the black color only for a short time, at most for not more than a day, when it returned with the same intensity as at first. Dr. Eulenberg prescribed some indifferent medicines, in order to keep the child under observation; directing the continued use of vinegar and water as an external application. But, notwithstanding the diarrhœa had long ceased, the tongue remained the same for three months. When the organ was cleansed, the black color reappeared, first in the middle and anterior half, afterwards gradually covering the entire dorsum of the tongue, and extending as far as could be seen. The *lingual papillæ* were, at the same time, much developed. The *papillæ filiformes* were very distinct, and were especially dark colored. The *papillæ vallatæ*, projecting in a conical form, presented, particularly at their apices, a deep-black covering. Even after the tongue was washed these *papillæ* retained the color, and were merely surrounded by a pale border, owing to which the black hue of the apex was rendered the more striking. If the colored tongue was scraped, a viscid brownish mucus was obtained, which, under the microscope, exhibited a large quantity of thickened epithelial cells and granular pigment.

If the mucus thus scraped off were dried upon paper, there remained extremely delicate black or dark brown filaments, about as thick as a fine hair, and from $\frac{1}{4}$ to $\frac{1}{2}'''$ in length, or minute irregular plates of the same length and breadth. If the latter were divided, they frequently afforded minute, crisped particles, like fine down. Particles of the same kind, however, were often met with independently. Their elasticity was evidenced in this, that they often sprung away when an attempt was made further to subdivide them with needles.

Under the microscope they represented distinct, very much thickened, and brownish colored epithelium scales, among which, in the less dark, but somewhat transparent places, the pigment granules could be remarked. The latter, however, presented themselves with especial distinctness at the edges of the epithelium scales, and appeared as irregular, rounded, flat granules, the border of which was dark, and the centre always clearer, but no nucleus was ever remarked in them. In the centre of the epithelium scales they occasionally constituted a beautiful mosaic area of rounded, closely approximated, elongated, or sub-angular granules. Punctiform granules were more rarely met with. The moniliform arrangement of the granules was remarked more especially at the border of the epithelium scales. The more transparent the epithelium, the more transparent, also, were the separate granules which then occurred isolated. The author seldom noticed a single isolated granule, for, however few might be connected together, they were usually supported on a small particle of an epithelium cell. When free, they were rounded or punctiform, and appeared connected in the form of a small rod or coronal. This description of the granular pigment does not agree, in all respects, with those given by other authors, as J. Vogel and Höfle. According to Vogel ('Path. Anat.,' p. 159,) it consists of fine granular molecules of a brown or black color, which are most usually contained in cells of various form and size. Occasionally, it would appear, these pigment molecules occur free, particularly in the parenchyma of melanotic lungs. According to Höfle (Chemie *v.* Mikroskopie, p. 274,) the pigment corpuscles are characterized by the intense black color and almost immeasurable smallness of the constituent granules. According to him, they would seem never to be surrounded by a membrane, but frequently encompassed by a homogenous cortex, not differing from the substance connecting the granules together. Henle (Allg. Anat., p. 282,) is more inclined to the assumption of the cellular nature of the pigment corpuscles, as Schwann states that he has noticed a molecular motion of the pigment corpuscles within the cell, which Höfle, on the other hand, declares to be impossible, since molecular corpuscles can never perform any movements within a gelatinous substance.

In the case now in question, the author never observed a cell or membrane, since the pigment corpuscles rarely occurred in the free state, but were almost always deposited on, or among the epithelium. They most resembled the pigment corpuscles figured by Henle (l. c., tab. i., fig. 12, D.,) in which, also, the border is dark and the centre somewhat clearer. According to him they are 0.0005-0.0007‴ in the longest diameter, and about 1-4th as thick as long. Under a stronger power, Henle also noticed some as transparent as water. On some occasions, the author observed, on the borders of the epithelial scales, and connected with them, elastic fibres, distinctly characterized by their dichotomous mode of division. A few times he noticed filaments lying quite isolated, which very closely resembled the thallus-filaments figured by Henle (l. c., note, p. 29.) They were never connected with the epithelial cells, and exhibited perfectly cylindrical canals, without transverse septa, and beset externally with black points. The latter were in no case pigment corpuscles, as they were globular, which was particularly evident in the granules situated on the external borders, whilst the pigment granules were always flattened, however small they might be. Even in the almost punctiform pigment granules, the darker border could be distinguished under favorable conditions of illumination. It is well known that the granular pigment, besides its normal deposition in the *corpus ciliare*, in the pulmonary tissue, in the integuments, &c., is, for the most part, presented only in the most various pathological structures. In the tongue it has not yet been met with, especially to the extent in which it occurred in the present case. Höfle (l. c., p. 59,) observed in five cases, in the fur of the tongue occurring in the healthy condition, dark brownish bodies, partly of a cylindrical, partly of an irregular form, and with three or four times the circumference of the largest epithelial scales, thickly beset with granules, and internally containing a sort of medullary body. He regarded this as an epithelial investment of the lingual papillæ. These cases would seem to present no similarity with the instance observed by the author. Höfle could never effect a division of these bodies into separate epithelial scales, which Dr. Eulenberg could always succeed in doing; nor could the latter ever observe the so-called medullary body; and, as regards the granules, Höfle describes them as black, scattered points, whilst in the author's case, they were aggregated in many ways, and represented roundish or angular and flattened granules or plates, with dark borders, and, much more rarely, simple points. The dark epithelial scales upon which the pigment corpuscles were chiefly deposited, the author also regards as an epithelial covering of the *papillæ*.

After he had observed the progress of the case for a sufficient length of time, and found that the phenomena remained unchanged, the author directed the internal use of an aqueous solution of chlorine, to be administered every three hours. After about two ounces of this medicament had been thus taken, not a vestige of the black coloration remained. The tongue had resumed its normal appearance, and the *papillæ* more of their natural size. The color had not recurred, even at the end of a year, so that the cure seemed to be complete.—*London Quar. Jour. of Mic. Science.*

